# Cod-Liver Oil in Scrofulous Affections and in Consumption

**Published:** 1851-12

**Authors:** 


*Cod-liver Oil in Scrofulous Affections and in Consumption.*— Dr. Hays stated to the Philadelphia College of Physicians, (Feb. 6, 1851,) that he had employed the cod-liver oil extensively, in the Wills' Hospital and in private practice, during the last three years, in scrofulous ophthalmia, in cases of granular lids, in scrofulous enlargement of the external glands, in cases of hip disease, and in the various forms of external scrofula, with the best effects. In scrofulous ophthalmia he had found it of all remedies the most efficacious. Under its use the constitution becomes invigorated; the glandular swellings are dissipated: and the cutaneous affection so commonly met with about the face and ears, disappears. He has also employed it in several cases of granular lids with the most favorable results. In this affection, patients are very liable to relapse, from slight causes; this tendency he has found to be removed by the use of the cod-liver oil, alone, or in conjunction with the syrup of the proto-iodide of iron. In the case of a lad now under treatment, affected with scrofulous enlargement of the cervical glands, chronic conjunctivitis, and granular lids, with deposit of lymph in the cornea, and intense photophobia, by the use of the cod-liver oil and proto-iodide of iron, with the occasional application to the eye of the liquor plumbi, all the symptoms of disease are rapidly disappearing. The patient can bear the light without inconvenience, can read small print, and has all the general appearance of restored health. He has escaped a relapse now for four months. The photophobia has disappeared entirely. In another case of excessive photophobia, with granular lids, and penetrating ulcer of the cornea, the cod-liver oil has been used (at the suggestion of the *interne* of the Wills' Hospital, Dr. McIntyre,) with the most decided advantage.

Dr. H. has now employed the cod-liver oil in from two hundred to two hundred and fifty cases of scrofulous ophthalmia and granular lids, and in most of these cases the benefit resulting from its use has been very striking. —*American Journal of the Medical Sciences.*

